# Effects of Kisspeptin-10 on Hypothalamic Neuropeptides and Neurotransmitters Involved in Appetite Control

**DOI:** 10.3390/molecules23123071

**Published:** 2018-11-24

**Authors:** Giustino Orlando, Sheila Leone, Claudio Ferrante, Annalisa Chiavaroli, Adriano Mollica, Azzurra Stefanucci, Giorgia Macedonio, Marilisa Pia Dimmito, Lidia Leporini, Luigi Menghini, Luigi Brunetti, Lucia Recinella

**Affiliations:** Department of Pharmacy, “G. d’Annunzio” University, Via dei Vestini 31, 66100 Chieti, Italy; giustino.orlando@unich.it (G.O.); sheila.leone@unich.it (S.L.); annalisa.chiavaroli@unich.it (A.C.); adriano.mollica@unich.it (A.M.); azzurra.stefanucci@unich.it (A.S.); giorgia.macedonio@unich.it (G.M.); marilisa.dimmito@unich.it (M.P.D.); lidia.leporini@unich.it (L.L.); luigi.menghini@unich.it (L.M.); luigi.brunetti@unich.it (L.B.); lucia.recinella@unich.it (L.R.)

**Keywords:** kisspeptin-10, neuropeptide Y, brain-derived neurotrophic factor, dopamine, 5-hydroxytriptamine

## Abstract

Besides its role as key regulator in gonadotropin releasing hormone secretion, reproductive function, and puberty onset, kisspeptin has been proposed to act as a bridge between energy homeostasis and reproduction. In the present study, to characterize the role of hypothalamic kisspeptin as metabolic regulator, we evaluated the effects of kisspeptin-10 on neuropeptide Y (NPY) and brain-derived neurotrophic factor (BDNF) gene expression and the extracellular dopamine (DA), norepinephrine (NE), serotonin (5-hydroxytriptamine, 5-HT), dihydroxyphenylacetic acid (DOPAC), and 5-hydroxyindoleacetic acid (5-HIIA) concentrations in rat hypothalamic (Hypo-E22) cells. Our study showed that kisspeptin-10 in the concentration range 1 nM–10 μM was well tolerated by the Hypo-E22 cell line. Moreover, kisspeptin-10 (100 nM–10 μM) concentration independently increased the gene expression of NPY while BDNF was inhibited only at the concentration of 10 μM. Finally, kisspeptin-10 decreased 5-HT and DA, leaving unaffected NE levels. The inhibitory effect on DA and 5-HT is consistent with the increased peptide-induced DOPAC/DA and 5-HIIA/5-HT ratios. In conclusion, our current findings suggesting the increased NPY together with decreased BDNF and 5-HT activity following kisspeptin-10 would be consistent with a possible orexigenic effect induced by the peptide.

## 1. Introduction

Kisspeptin is a peptide encoded by the metastasis suppressor gene kiSS-1 for melanoma cells [1], isolated from human placenta and acting as endogenous ligand of the orphan G protein-coupled receptor 54 (GPR54, now named kisspeptin receptor, kiSS-1R) [2,3,4]. The kiSS-1 gene product is cleaved to produce 54-, 14-, 13-, and 10-amino acid peptides, with a common RF-amide C terminus, all of which activate the receptor and possess biological activity [2,4,5]. Kisspeptin has been identified in multiple brain areas including the hippocampus and amygdala [6,7,8]. Additionally, kisspeptin neurons are widely distributed in many discrete hypothalamic nuclei in both mouse and rat, including the anteroventral periventricular nucleus, preoptic periventricular nucleus, and arcuate nucleus (ARC) [9,10,11]. The ARC contains a high density of orexigenic neuropeptide Y (NPY) and agouti-related peptide (AgRP) co-expressing neurons, as well as anorexigenic proopiomelanocortin (POMC) and cocaine- and amphetamine-regulated transcript (CART) peptide co-expressing neurons [12]. NPY/AgRP neurons and POMC/CART peptide neurons from ARC project to the lateral hypothalamus, modulating orexin containing neurons which increase food intake, and to the paraventricular nucleus (PVN), modulating corticotrophin releasing hormone (CRH) neurons which decrease feeding [12].

Kisspeptin and GPR54 are known as key regulators in gonadotropin releasing hormone (GnRH) secretion, reproductive function, and puberty onset [13]. Kisspeptin has also been proposed to act as a bridge between energy homeostasis and reproduction [14]. To this regard, kisspeptin and its analog TAK-683 have been reported to stimulate luteinizing hormone release in vivo [15,16]. Moreover, central, but not peripheral, injection of kisspeptin-10 was found to decrease food intake in overnight fasted mice [5]. However, kisspeptin neurons are direct targets for regulation by leptin which could act at a post-transcriptional level [17], and it is well known that leptin plays a pivotal role in the hypothalamic regulation of feeding behavior, energy homeostasis, and reproduction [18,19]. Conversely, central injection of kisspeptin-10 was not found to affect feeding in rats [16,17]. Nevertheless, kisseptin gene resulted in upregulation in female rats fed on cafeteria diet, further supporting a discrete role of kisspeptin in energy balance control [20]. Finally, food deprivation or other conditions of negative energy balance, including chronic calorie restriction, lead to a significant reduction in hypothalamic kiSS-1 mRNA levels in rats [17,21].

The role of NPY as a potent orexigenic agent in the hypothalamus has been well characterized, and food restriction has been shown to increase NPY mRNA [22] and NPY immunoreactivity [23] in the ARC. Additionally, kisspeptin could stimulate growth hormone release via NPY signaling [24]. Several studies suggested that brain-derived neurotrophic factor (BDNF) is also involved in feeding behavior and body weight regulation, with anorexigenic effects. BDNF is expressed in the hypothalamus, particularly in feeding regulatory areas, including ventromedial hypothalamus, dorsomedial nucleus of the hypothalamus, paraventricular nucleus of the hypothalamus, and the ARC [25].

Biogenic amines are also known as critical modulators of feeding behavior at the hypothalamic level. Accordingly, our previous studies showed that adipocyte- and gut-derived hormones which are known to affect feeding displayed inhibitory or stimulatory effects on hypothalamic dopamine (DA), norepinephrine (NE), and serotonin (5-hydroxitriptamine, 5-HT) levels [26,27,28,29].

In the present study, to further characterize the role of hypothalamic kisspeptins as metabolic regulators, we evaluated the effects of kisspeptin-10 on NPY and BDNF gene expression and the extracellular DA, NE, and 5-HT concentrations in rat hypothalamic (Hypo-E22) cells. Additionally, we measured the levels of dihydroxyphenylacetic acid (DOPAC) and 5-hydroxyindoleacetic acid (5-HIIA), which are the main metabolites of DA and 5-HT, respectively.

## 2. Results

### Pharmacological Studies

MTT test showed that kisspeptin-10 in the concentration range 1 nM–10 μM did not affect Hypo-E22 cell line viability (data reported in Supplementary Material).

**Figure 1:**
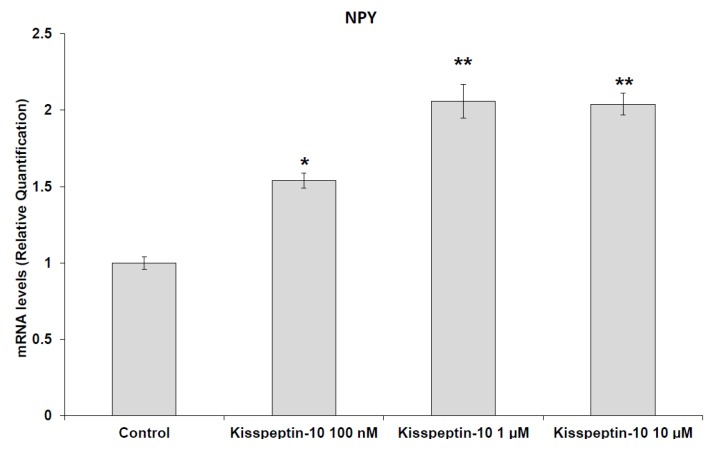
Effect of kisspeptin-10 (100 nM–10 μM) treatment on relative gene expression of neuropeptide Y (NPY), as determined by real-time RT PCR in rat hypothalamic (Hypo-E22) cell line (*n* = 5 individual culture flasks for each condition). Data were calculated using the 2^−ΔΔCt^ method; they were normalized to β-actin mRNA levels and then expressed as relative to vehicle. Compared to control, kisspeptin-10 treatment significantly increased NPY (ANOVA, *p* < 0.001; post-hoc, * *p* < 0.05, ** *p* < 0.001 vs. control) gene expression.

**Figure 2:**
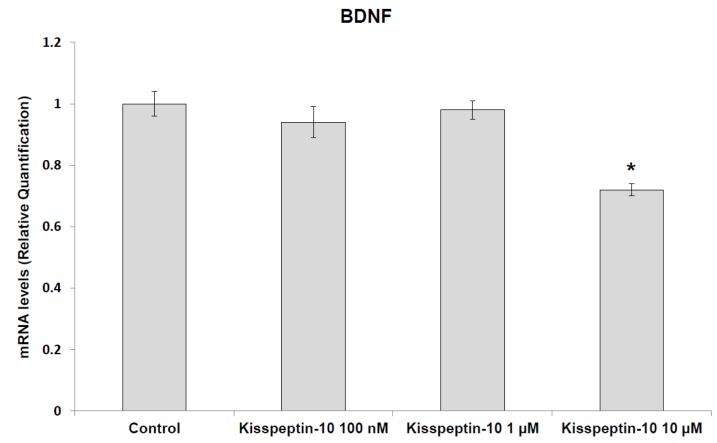
Effect of kisspeptin-10 (100 nM–10 μM) treatment on relative gene expression of brain-derived neurotrophic factor (BDNF), as determined by real-time RT PCR in rat hypothalamic (Hypo-E22) cell line (*n* = 5 individual culture flasks for each condition). Data were calculated using the 2^−ΔΔCt^ method; they were normalized to β-actin mRNA levels and then expressed as relative to vehicle. Compared to control, kisspeptin-10 treatment significantly decreased BDNF (ANOVA, *p* < 0.01; post-hoc, * *p* < 0.05 vs. control) gene expression.

Additionally, Kisspeptin-10 (100 nM–10 μM) stimulated the gene expression of NPY while decreasing BDNF gene expression (Figure 1 and Figure 2).

**Figure 3:**
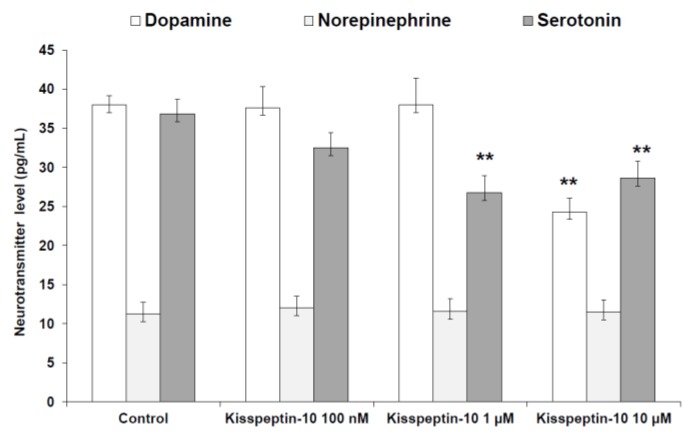
Effect of kisspeptin-10 (100 nM–10 μM) treatment on extracellular dopamine (DA), norepinephrine (NE), and serotonin (5-hydroxytriptamine, 5-HT) levels, as determined by HPLC in rat hypothalamic (Hypo-E22) cell line (*n* = 5 individual culture flasks for each condition). Compared to control, kisspeptin-10 treatment significantly decreased extracellular DA (ANOVA, *p* < 0.01; post-hoc, ** *p* < 0.01 vs. control) and 5-HT (ANOVA, *p* < 0.01; post-hoc, ** *p* < 0.01 vs. control) levels.

**Figure 4:**
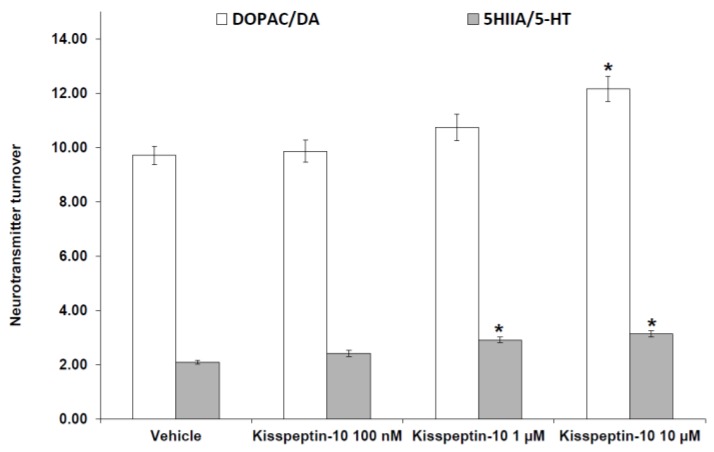
Effect of kisspeptin-10 (100 nM–10 μM) treatment on extracellular dihydroxyphenylacetic acid (DOPAC)/dopamine (DA) and 5-hydroxyindoleacetic acid (5-HIIA)/serotonin (5-HT) ratios as determined by HPLC in rat hypothalamic (Hypo-E22) cell line (*n* = 5 individual culture flasks for each condition). Compared to control, kisspeptin-10 treatment significantly increased extracellular DOPAC/DA and 5-HIIA/5-HT ratios (ANOVA, *p* < 0.01; post-hoc, * *p* < 0.05 vs. control).

Kisspeptin-10 (100 nM–10 μM) treatment decreased 5-HT and DA, leaving unaffected NE levels (Figure 3). The inhibitory effect on extracellular DA and 5-HT is consistent with the stimulated DOPAC/DA and 5-HIIA/5-HT ratios, following peptide treatment (Figure 4).

## 3. Discussion

Our study revealed that kisspeptin-10 concentration-independently increased the gene expression of NPY (Figure 1) while BDNF was inhibited only at the concentration of 10 μM (Figure 2). NPY and kisspeptin neurons are known to play a key role in the neuronal network involved in the control of GnRH pulsatile release. Moreover, NPY has been proposed to be a potential metabolic regulator of kisspeptin neurons. Consistently, previous studies have demonstrated that NPY neurons are in close proximity to kisspeptin neuronal projections in the ARC of the hypothalamus [30], and kiSS-1R is expressed in hypothalamic NPY neurons [31]. Notably, Luque and colleagues [32] demonstrated that exposure to NPY significantly increases kiSS-1 mRNA levels in immortalized hypothalamic cell line N6. This is consistent with in vivo studies on NPY-knockout mice showing downregulated kiSS-1 gene expression. Furthermore, kisspeptin-10 treatment was found to increase immunoreactivity of the immediate early gene protein c-Fos in NPY neurons of the hypothalamus of fasted sheep [24]. In this context, the elevation in NPY mRNA levels after kisspeptin-10 treatment (Figure 1) further support the hypothesis that kisspeptin stimulates NPY release by activation of NPY neurons in the ARC. Previously, Fu and van den Pol [33] suggested an indirect inhibitory interaction of NPY activity by kisspeptin, possibly mediated by stimulated POMC signaling. This is consistent with the reciprocal inhibitory activity of POMC and NPY neurons in the hypothalamus [34]. Our contrasting result, which is conversely consistent with the work by Luque and colleagues [32], could depend on the null expression of POMC gene in our cell line (data not reported), which could blunt the inhibitory effect observed by Fu and van den Pol [33]. Besides its role on neuronal development and synaptic plasticity modulation [35], BDNF has been reported to play a critical role in regulating energy balance. Central injection of BDNF was shown to decrease feeding and increase energy expenditure, with a reduction in body weight gain [36,37]. Moreover, BDNF has been shown to be a critical effector by which melanocortin-4 receptor-mediated signaling regulates energy balance [38]. Our findings of reduced BDNF and increased NPY gene expression could suggest a possible orexigenic effect of kisspeptin-10 in the hypothalamus. By contrast, previous studies on the role of kisspeptin-10 in the control of food intake have been inconsistent. Kisspeptin-10 was reported to play an anorexigenic role in the central modulation of feeding in mice [5], while no effects on food intake was found in rats [16,17]. In order to further investigate the potential involvement of kisspeptin-10 in central appetite regulation, we evaluated extracellular DA, NE, and 5-HT in the Hypo-E22 cells incubated with kisspeptin-10 (100 nM–10 μM). We found that kisspeptin-10 treatment decreased 5-HT and DA, leaving unaffected NE levels (Figure 3). Consistent with these findings, kisspeptin-10 also stimulated DOPAC/DA and 5-HIIA/5-HT ratios (Figure 4), which have been long considered as valuable indices of DA and 5-HT turnover, respectively [39]. Regarding extracellular DA level, the inhibitory effect was observed only at the highest concentration of the peptide (10 μM). Accordingly, kisspeptin-10 was reported to increase prolactin release by inhibiting dopaminergic neurons in rat hypothalamus, showing a close apposition between kisspeptin fibers and dopaminergic neurons in the ARC [40]. The role of DA in central feeding behavior modulation has been well documented, with both stimulatory and inhibitory effects. In keeping with the latter, administration of DA in the perifornical area of the rat hypothalamus inhibited food intake [41]. By contrast, DA injection into the lateral hypothalamus (LH) was able to increase feeding and higher levels of DA in the LH were found in obese rats [42]. Furthermore, our previous studies demonstrated that anorexigenic peptides such as obestatin and irisin inhibit dopamine release in rat hypothalamus [26,29]. On the other hand, the critical role of serotonin at the hypothalamic level in feeding inhibition is well known [43] and we have previously reported that orexigenic peptides, such as apelin-13, inhibited hypothalamic serotonin synthesis and release, which could be related to its effects on feeding [28]. Recent findings showed possible relationships between appetite-stimulating hormones and hypothalamic pathways involved in reproduction and growth. Specifically, we have demonstrated that the orexigenic peptide ghrelin was able to inhibit 5-HT level in the hypothalamus of growth hormone releasing hormone (GHRH) knock out mice [44].While mutations on kisspeptin signaling have been related to precocious puberty (CPP) and idiopathic hypogonadotropic hypogonadism [45].

In conclusion, our current findings showing the increased NPY together with decreased BDNF gene expression and 5-HT level following kisspeptin-10 treatment would be consistent with a possible orexigenic effect induced by the peptide. Finally, considering that 5-HT signaling has been involved in both appetite-suppressing effect and hypogonadism [46], the inhibitory effect induced by kisspeptin-10 on 5-HT level further supports a possible role of the peptide in both appetite and reproduction regulating pathways. 

## 4. Materials and Methods

### 4.1. Chemistry

Kisspeptin-10 (H-Tyr-Asn-Trp-Asn-Ser-Phe-Gly-Leu-Arg-Phe-NH_2_, YNWNSFGLRF-NH_2_) (structure reported as Supplementary Materials) has been prepared in our laboratory by Fmoc-solid phase peptide synthesis (Fmoc-SPPS) strategy on Rink amide resin, following standard Fmoc-strategy by using TBTU/HOBt for coupling reactions and piperidine 20% solution in DMF for Fmoc group deprotection [47,48,49]. Fmoc-Tyr(*t*Bu)-OH, Fmoc-Trp(Boc)-OH, Fmoc-Arg(Pbf)-OH, Fmoc-Asn(Trt)-OH, and Fmoc-Ser(*t*Bu)-OH were used as orthogonally protected building blocks for coupling reactions [47,50].

Solvents and reagents were purchased from Sigma-Aldrich (Milan, Italy) and used as supplied without further purification. Amino acids were purchased from Iris Biotech GMBH (Marktredwitz, Germany) and Sigma-Aldrich (Italy).

Chromatographic purification was performed by RP-HPLC semipreparative C18 column (eluent: ACN/H_2_O gradient, 5–95% over 32 min) at a flow gradient of 4 mL/min. Kisspeptin-10 was characterized by ^1^H-NMR spectra on 300 MHz Varian Inova spectrometer (Varian Inc., Palo Alto, CA, USA); chemical shifts are reported in parts per million downfield from the internal standard tetramethylsilane (Me4Si). Mass spectra was performed on Thermo Scientific Q Exactive (Thermo Fisher Scientific, San Jose, CA, USA), in the positive mode, capillary temperature 220 °C, spray voltage 2.3 kV, and sheath gas 5 units.

### 4.2. Synthesis and Characterization

The resin was treated with 20% piperidine solution in DMF (2 × 15 min) and then washed with DMF/MeOH/DCM. The first Fmoc-Xaa-OH (3 equiv.) was dissolved in DMF (3 mL). TBTU (3 equiv.) and DIPEA (6 equiv.) were added and the resulting mixture was added to the resin, and stirred overnight. The Kaiser test was used to check the reaction. When complete, the resin was washed with DMF/MeOH/DCM, Fmoc group was removed with a solution of piperidine 20% in DMF before coupling cycles (Scheme S1 in supplementary material).

Fmoc-AA-OH (3 equiv.) and HOBT (3 equiv.) were dissolved in DMF (6 mL). TBTU (3 equiv.) and DIPEA (6 equiv.) were added and the resulting mixture was added to the resin. The Kaiser test was used to check the reaction. When complete, the resin was washed with DMF/MeOH/DCM. In place of DIPEA a solution of collidine was used as base for coupling with the following amino acids: Fmoc-Trp(Boc)-OH, Fmoc-Asn(trt)-OH, and Fmoc-Tyr(*t*Bu)-OH. Following the peptide’s elongation, the resin was treated with a cocktail of TFA/H_2_O/TIPS = 95:2.5:2.5 (5 mL for 1 h). The resin was filtered off and the volume of the solution reduced to 1 mL at rotary evaporator, then it was precipitated in 10 mL of cold Et_2_O. The suspension was centrifuged and washed 3 times with fresh Et_2_O, then the desired crude peptide was dried in high vacuum. The crude peptide was purified on RP-HPLC to yield the desired white product in 38% yield (Rt 15.53 min), HRMS calcd.: 1302.4620 g/mol, found: 1303.4628 [M + H]. ^1^H-NMR (DMSO-*d*_6_): 10.73 (1H, s, NH Trp indole), 9.32 (1H, s, OH Tyr), 9.16 (1H, d, NH Tyr), 8.68–7.81 (7H, m, NH backbone), 7.53–6.78 (27H, m, 3NH + 16 aromatics + 8NH_2_), 6.78 (2H, dd, Tyr aromatics), 4.93 (1H, t, CH Gly), 4.59–3.71 (10H, m, CH amino acids), 2.86–2.54 (8H, m, CH amino acids), 1.71 (2H, m, CH Arg), 1.38 (2H, m, CH Arg), 0.67 (6H, sept, CH3 Leu).

### 4.3. Cell Cultures and Viability Test

Hypo-E22 cells were purchased from Cedarlane Cellution Biosystem and cultured in DMEM (Euroclone) supplemented with 10% (*v*/*v*) heat-inactivated fetal bovine serum and 1.2% (*v*/*v*) penicillin G/streptomycin in 75 cm^2^ tissue culture flask (*n* = 5 individual culture flasks for each condition). The cultured cells were maintained in a humidified incubator with 5% CO_2_ at 37 °C. For cell differentiation, cell suspension at a density of 1 × 106 cells/mL was treated with various concentrations (10, 50, and 100 ng/mL) of phorbol myristate acetate (PMA, Fluka) for 24 h or 48 h (induction phase). Thereafter, the PMA-treated cells were washed twice with ice-cold pH 7.4 phosphate buffer solution (PBS) to remove PMA and non-adherent cells, whereas the adherent cells were further maintained for 48 h (recovery phase). Morphology of cells was examined under an inverted phase-contrast microscope [51]. To assess the basal cytotoxicity of kisspeptin, a viability test was performed on 96 microwell plates, using 3-(4,5-dimethylthiazol-2-yl)-2,5-diphenyltetrazolium bromide (MTT) test. Cells were incubated with kisspeptin-10 (ranging concentration 1 nM–10 μM) [52,53,54,55] for 24 h. After the treatment period, 10 μL of MTT (5 mg/mL) were added to each well and incubated for 3 h. The formazan dye formed was extracted with dimethyl sulfoxide and absorbance recorded as previously described [56]. Effects on cell viability were evaluated in comparison to untreated control group. Following viability tests, Hypo-E22 cells were treated with kisspeptin-10 (1 nM–10 μM) for 24 h (stimulation). At the end of the stimulation period, we evaluated extracellular DA, NE, and 5-HT levels and BDNF and NPY gene expression, as previously described [57,58].

### 4.4. RNA Extraction, Reverse Transcription, and Real-Time Reverse Transcription Polymerase Chain Reaction (Real-Time RT PCR)

Total RNA was extracted from the cells using TRI Reagent (Sigma-Aldrich, St. Louis, MO, USA), according to the manufacturer’s protocol. Contaminating DNA was removed using 2 units of RNase-free DNase 1 (DNA-free kit, Ambion, Austin, TX, USA). The RNA solution was quantified at 260 nm by spectrophotometer reading (BioPhotometer, Eppendorf, Hamburg, Germany) and its purity was assessed by the ratio at 260 and 280 nm readings. The quality of the extracted RNA samples was also determined by electrophoresis through agarose gels and staining with ethidium bromide under UV light. 1 μg of total RNA was reverse transcribed using High Capacity cDNA Reverse Transcription Kit (Applied Biosystems, Foster City, CA, USA). Reactions were incubated in a 2720 Thermal Cycler (Applied Biosystems, Foster City, CA, USA) initially at 25 °C for 10 min, then at 37 °C for 120 min, and finally at 85 °C for 5 s. Gene expression was determined by quantitative real-time PCR using TaqMan probe-based chemistry (Applied Biosystems, Foster City, CA, USA). PCR primers and TaqMan probes were obtained from Applied Biosystems (Assays-on-Demand Gene Expression Products, Rn02531967_s1 for BDNF gene; Rn00561681_m1 for NPY gene). β-actin (Applied Biosystems, Foster City, CA, USA, Part No. 4352340E) was used as the housekeeping gene. The real-time PCR was carried out in triplicate for each cDNA sample in relation to each of the investigated genes. Data were elaborated with the Sequence Detection System (SDS) software version 2.3 (Applied Biosystems, Foster City, CA, USA).

### 4.5. Neurotransmitter Extraction and High Performance Liquid Chromatography (HPLC) Determination

Extracellular DA, 5-HT, and NE levels were analyzed through an HPLC apparatus consisting of a Jasco (Tokyo, Japan) PU-2080 chromatographic pump and an ESA (Chelmsford, MA, USA) Coulochem III coulometric detector, equipped with microdialysis cell (ESA-5014b) porous graphite working electrode and solid state palladium reference electrode. The analytical conditions for biogenic amine identification and quantification were selected as previously reported [40]. Briefly, the analytical cell was set at −0.150 V for detector 1 and at +0.300 V for detector 2, with a range of 100 nA. The chromatograms were monitored at the analytical detector 2. Integration was performed by Jasco Borwin Chromatography software, version 1.5. The chromatographic separation was performed by isocratic elution on Phenomenex Kinetex reverse phase column (C18, 150 mm × 4.6 mm i.d., 2.6 µm). The mobile phase was (10:90, *v*/*v*) acetonitrile and 75 mM pH 3.00 phosphate buffer containing octanesulfonic acid 1.8 mM, EDTA 30 µM, and triethylamine 0.015% *v*/*v*. Flow rate was 0.6 mL/min and the samples were manually injected through a 20 µL loop. Neurotransmitter peaks were identified by comparison with the retention time of pure standard. Neurotransmitter concentrations in the samples were calculated by linear regression curve (y = bx + m) obtained with standard. Neither internal nor external standard were necessary for neurotransmitter quantification in the hypothalamus homogenate, and all tests performed for method validation yielded results in accordance to limits indicated in official guidelines for applicability in laboratory trials (See Supplementary Materials). The standard stock solutions of DA, NE, 5-HT, DOPAC, and 5-HIIA at 2 mg/mL were prepared in bidistilled water containing 0.004% EDTA and 0.010% sodium bisulfite. The stock solutions were stored at 4 °C. Work solutions (1.25–20.00 ng/mL) were obtained daily, progressively diluting stock solutions in mobile phase.

### 4.6. Statistical Analysis

Statistical analysis was performed using GraphPad Prism version 5.01 for Windows (GraphPad Software, San Diego, CA, USA). Means ± SEM were determined for each experimental group and analyzed by one-way analysis of variance (ANOVA) followed by Newman-Keuls comparison multiple test. Statistical significance was set at *p* < 0.05. As regards to gene expression analysis, the comparative 2^−ΔΔCt^ method was used to quantify the relative abundance of mRNA and then to determine the relative changes in individual gene expression (relative quantification) [59].

## Supplementary Materials

The following are available online. Figure S1 a: Kisspeptin structure; Figure S2 MTT: cell viability; Tables S1–S4: HPLC-EC method; Scheme S1: Kisspeptin 10 synthesis.
