# Supplementary material for: Effects of Kisspeptin-10 on Hypothalamic Neuropeptides and Neurotransmitters Involved in Appetite Control
Source: Molecules. 2018 Nov 24;23(12):3071. doi: 10.3390/molecules23123071 (PMC6321454; doi:10.3390/molecules23123071)
Supplement: Supplementary file 1 [file molecules-23-03071-s001.pdf]

Table S1

	Standard concentrations			
	**2.5 (ng/ml)**	**5.0 (ng/ml)**	**10 (ng/ml)**	**20.0 (ng/ml)**
**NE**	**1.79**	**2.58**	**1.39**	**0.51**
**DA**	**0.34**	**7.10**	**5.32**	**2.88**
**5-HT**	**1.48**	**3.84**	**0.10**	**0.34**

Table S1: Percentage relative standard deviation (RSD%) obtained from analysis of standard solutions of norepinephrine (NE), dopamine (DA) and serotonin (5-HT).

Table S2

**NE, DA, 5-HT**	**1.25 (ng/ml)**	**3.75 (ng/ml)**	**11.00 (ng/ml)**	**15.00 (ng/ml)**
	**Resulting concentration calculated by linear regression curve**			
**NE, DA, 5-HT**	**1.25, 1.24, 1.24**	**3.93, 3.85, 3.84**	**10.99, 11.25, 10.97**	**15.08, 15.03, 15.18**
	**Percentage variation**			
**NE, DA, 5-HT**	**0, 0.8, 0.8**	**-4.8, -2.7, -2.4**	**0.1, -2.3, 0.3**	**-0.5, -0.2, -1.2**

Table S2: Mean percentage variations of standard solutions of norepinephrine (NE), dopamine (DA) and serotonin (5-HT), at four different concentrations calculated by linear regression curve.

Table S3

		Sample dilution	RSD%	Mean concentration (ng/ml)	Recovery %
NE	LOQ	1:50	7.42	0.935	98.92
NE	LOD	1:100	75.74	0.408	86.28
DA	LOQ	1:15	0.11	1.228	98.40
DA	LOD	1:30	27.13	0.690	111.01
5-HT	LOQ	1:100	3.62	1.295	101.27
5-HT	LOD	1:200	23.50	0.561	87.76

Table S3: The limits of quantitation (LOQ) and detection (LOD) of norepinephrine (NE), dopamine (DA) and serotonin (5-HT) were determined by analyses performed in triplicate on hypothalamic homogenate serially diluted in mobile phase. The LOQ is defined, for each analyte, as the lowest concentration showing a percentage relative standard deviation (RSD%) ≤10.

Table S4

		DA	NE	5-HT
**10 ng/ml**	**RSD%**	**2.74**	**1.92**	**1.61**
	**Mean concentration (ng/ml)**	**9.57**	**9.24**	**9.81**
**10.5 ng/ml**	**RSD%**	**1.07**	**1.89**	**1.52**
	**Mean concentration (ng/ml)**	**10.42**	**10.39**	**10.30**
**11 ng/ml**	**RSD%**	**1.02**	**0.48**	**0.65**
	**Mean concentration (ng/ml)**	**11.27**	**11.26**	**11.29**

Table S4: Sensitivity analysis, performed by five injections of three standard solutions of norepinephrine (NE), dopamine (DA) and serotonin (5-HT), reveals that our method discriminates concentration differences of magnitude 0.5 ng/ml, with a percentage relative standard deviation (RSD%) ≤10.

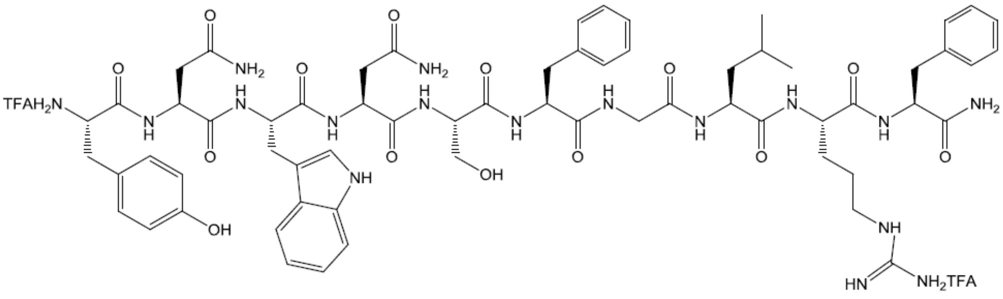

Figure S1: Structure of Kisspeptin-10.

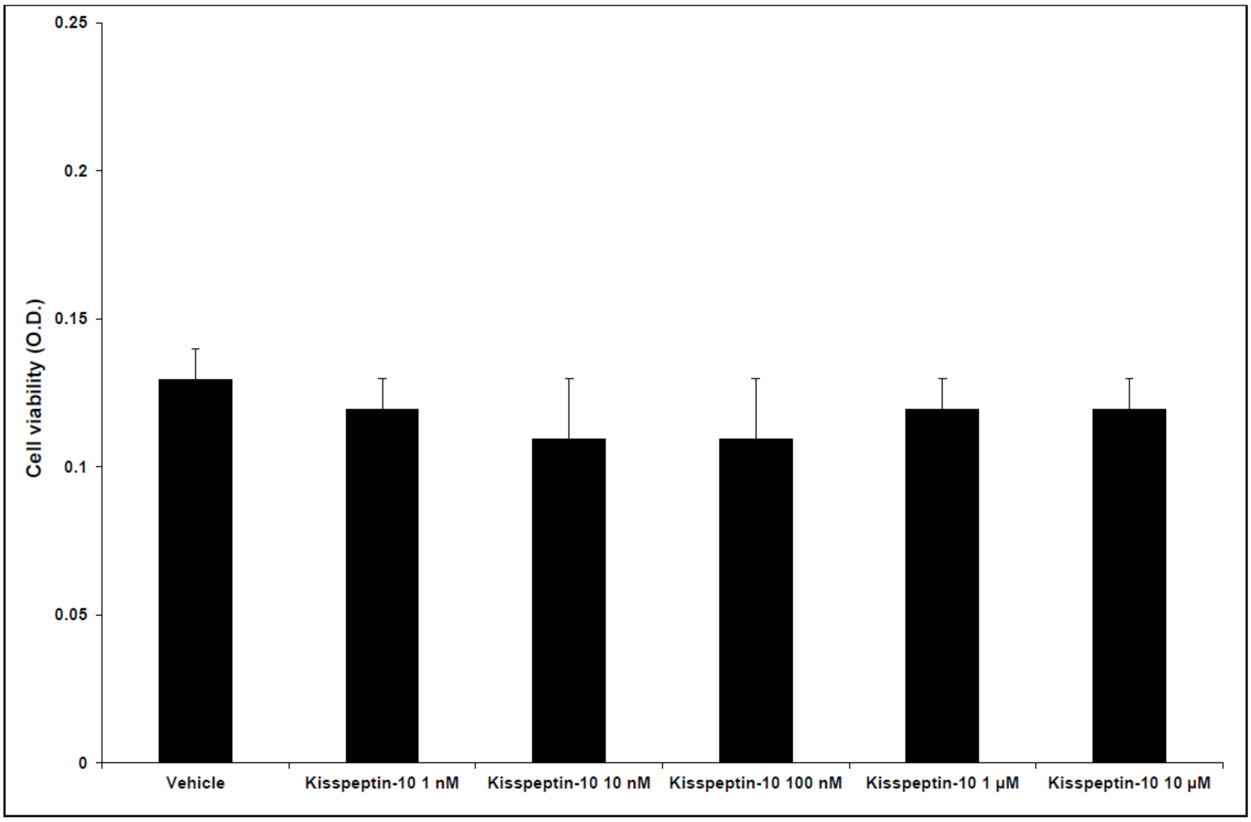

Figure S2: Effect of Kisspeptin-10 1nM-10 μM on hypothalamic hypoE22 cell line (MTT viability test).

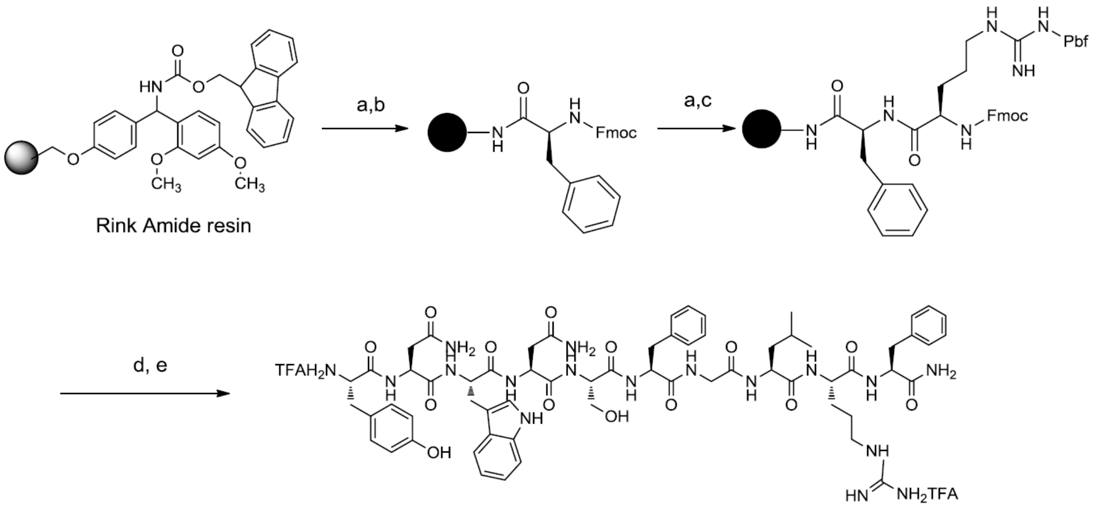

Scheme S1: Synthesis of Kisspeptin-10. Reagents and conditions: a. Piperidine 20% in DMF; b. Fmoc-Phe-OH, HOBt/TBTU, DIPEA, DMF; c. Fmoc-Arg(Pbf)-OH, HOBt/TBTU, DIPEA, DMF; d. cycles of Fmoc-deprotection/repeated couplings; e. TFA/H2O/TIPS 95:2.5:2.5.
